# Oncolytic Reovirus-Mediated Recruitment of Early Innate Immune Responses Reverses Immunotherapy Resistance in Prostate Tumors

**DOI:** 10.1016/j.omto.2020.09.010

**Published:** 2020-10-04

**Authors:** Nicola E. Annels, Guy R. Simpson, Mick Denyer, Mehreen Arif, Matt Coffey, Alan Melcher, Kevin Harrington, Richard Vile, Hardev Pandha

**Affiliations:** 1 Targeted Cancer Therapy, Department of Clinical and Experimental Medicine, Leggett Building, University of Surrey, Guildford, Surrey GU2 7WG, UK; 2 Oncolytics Biotech, Inc., 210, 1167 Kensington Crescent NW Calgary, AB T2N 1X7, Canada; 3 Translational Immunotherapy Team, The Institute of Cancer Research, 237 Fulham Road, London SW6 6JB, UK; 4 Targeted Therapy Team, The Institute of Cancer Research, 237 Fulham Road, London SW6 6JB, UK; 5 Department of Immunology, Mayo Clinic, Rochester, MN 55905, USA

**Keywords:** Oncolytic virus, Prostate cancer, Immunotherapy resistance

## Abstract

Prostate cancers are considered “cold” tumors characterized by minimal T cell infiltrates, absence of a type I interferon (IFN) signature, and the presence of immunosuppressive cells. This non-inflamed phenotype is likely responsible for the lack of sensitivity of prostate cancer patients to immune checkpoint blockade (ICB) therapy. Oncolytic virus therapy can potentially overcome this resistance to immunotherapy in prostate cancers by transforming cold tumors into “hot,” immune cell-infiltrated tumors. We investigated whether the combination of intratumoral oncolytic reovirus, followed by targeted blockade of Programmed cell death protein 1 (PD-1) checkpoint inhibition and/or the immunomodulatory CD73/Adenosine system can enhance anti-tumor immunity. Treatment of subcutaneous TRAMP-C2 prostate tumors with combined intratumoral reovirus and anti-PD-1 or anti-CD73 antibody significantly enhanced survival of mice compared with reovirus or either antibody therapy alone. Only combination therapy led to rejection of pre-established tumors and protection from tumor re-challenge. This therapeutic effect was dependent on CD4^+^ T cells and natural killer (NK) cells. NanoString immune profiling of tumors confirmed that reovirus increased tumor immune cell infiltration and revealed an upregulation of the immune-regulatory receptor, B- and T-lymphocyte attenuator (BTLA). This expression of BTLA on innate antigen-presenting cells (APCs) and its ligand, Herpesvirus entry mediator (HVEM), on T cells from reovirus-infected tumors was in keeping with a role for the HVEM-BTLA pathway in promoting the potent anti-tumor memory response observed.

## Graphical Abstract

**Figure:**
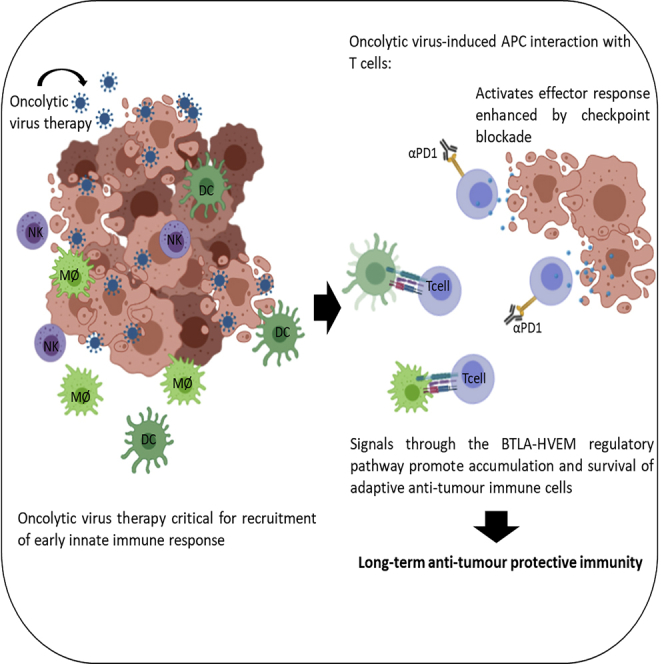

## Teaser

Immunotherapy in prostate cancer is limited because of a lack of immune cells within the tumor. Annels et al. used oncolytic viruses to recruit immune cells into the tumor and showed that in combination with checkpoint inhibitors, which take the brake off of the immune system, established tumors were rejected.

## Introduction

Although cancer immunotherapy with monoclonal antibodies (mAbs) against immune checkpoints has revolutionized the treatment of patients affected by certain cancer types,^1^^,^^2^ clinical trials in patients with prostate cancer have shown them to be less sensitive to this therapeutic approach.^3^, ^4^, ^5^ These findings have fueled significant research efforts to identify pre-existing and acquired mechanisms of resistance to immune checkpoint blockade (ICB). Factors that define sensitivity to cancer immunotherapies include tumor mutational burden^6^, ^7^, ^8^ and how “hot”/immune-infiltrated and consequently immunogenic a tumor is.^9^, ^10^, ^11^ Prostate cancers carry a lower mutational burden than other epithelial tumors^12^ and are generally considered to be “cold” tumors with minimal T cell infiltrates.

One approach to transform cold, immune-excluded prostate tumors into hot, immune-infiltrated ones is by exploiting the immune-stimulating properties of oncolytic viruses (OVs). Oncolytic reovirus is one of the most promising agents currently being investigated in the clinic. Like other OVs, reovirus is thought to mediate anti-tumor activity through a dual mechanism of selective replication within and lysis of infected cancer cells, while simultaneously inducing host anti-tumor immunity.^13^, ^14^, ^15^ Tumor lysis by OVs induces immunological “danger” signals and releases tumor antigens (immunogenic cell death [ICD]).^16^ Reovirus can also infect and activate dendritic cells (DCs) directly, which, upon their maturation, activate natural killer (NK) and T cells to kill cancer cells.^17^ The efficacy of reovirus as a therapeutic agent has been demonstrated by our group and others in numerous preclinical and clinical trials.^18^, ^19^, ^20^, ^21^, ^22^ Most importantly, intralesional reovirus injections in murine cancer models and human patients have resulted in an inflammatory effect with significant T cell infiltration. However, early-phase monotherapy clinical trials of reovirus have failed to demonstrate appreciable clinical efficacy in the form of objective and durable responses, highlighting the need to augment reovirus’s therapeutic potency.

Although reovirus infection clearly induces effector T cells to enter the tumor microenvironment, their phenotype and function will be affected by other immune and non-immune cells, as well as the physical properties of the tumor. Tumor-infiltrating lymphocytes (TILs) become dysfunctional because of their increased expression of inhibitory receptors, as well as the presence of immunosuppressive factors and regulatory cells.^23^ Furthermore, the prostate immune environment may change in response to treatment exposure. This was recently demonstrated in a clinical study using reovirus to treat high-grade glioma and brain metastases, where preconditioning of the tumor immune microenvironment by the virus upregulated tumor PD-L1 protein expression.^24^ Learning from these emerging published data, the existence of disease treatment-specific immune-inhibitory mechanisms needs to be investigated to improve the OV-initiated anti-tumor response.

Therefore, in the current study, we investigated whether the effectiveness of oncolytic viral therapy for prostate cancer could be improved with targeted blockade of PD-1 and/or CD73. Although PD-1 is a checkpoint receptor expressed on T cells, B cells, and monocytes,^25^ CD73 is an ecto-5′-nucleotidase that converts AMP to adenosine, an immune-suppressive molecule.^26^^,^^27^ Previous work has shown that targeted blockade of CD73 can enhance the therapeutic activity of anti-PD-1.^28^ Hence targeted blockade using antibodies against each molecule was performed individually or in combination with or without prior reovirus therapy. Our study using a preclinical model of subcutaneous TRAMP-C2 prostate revealed that oncolytic reovirus therapy was crucial for initial priming of immune effector cells upon which the anti-PD-1 or anti-CD73 antibody therapy could then act to further improve the anti-tumor immune response. Further immune profiling of reovirus-treated TRAMP-C2 tumors revealed the upregulation of BTLA, which rather than acting as an inhibitory receptor, appeared to have a critical role in HVEM-BTLA co-signaling *in trans*, promoting the optimal generation of a potent anti-tumor memory response.

## Results

### TRAMP-C2 Cells Are Highly Susceptible to *In Vitro* Reovirus-Induced Oncolysis

**Figure 1:**
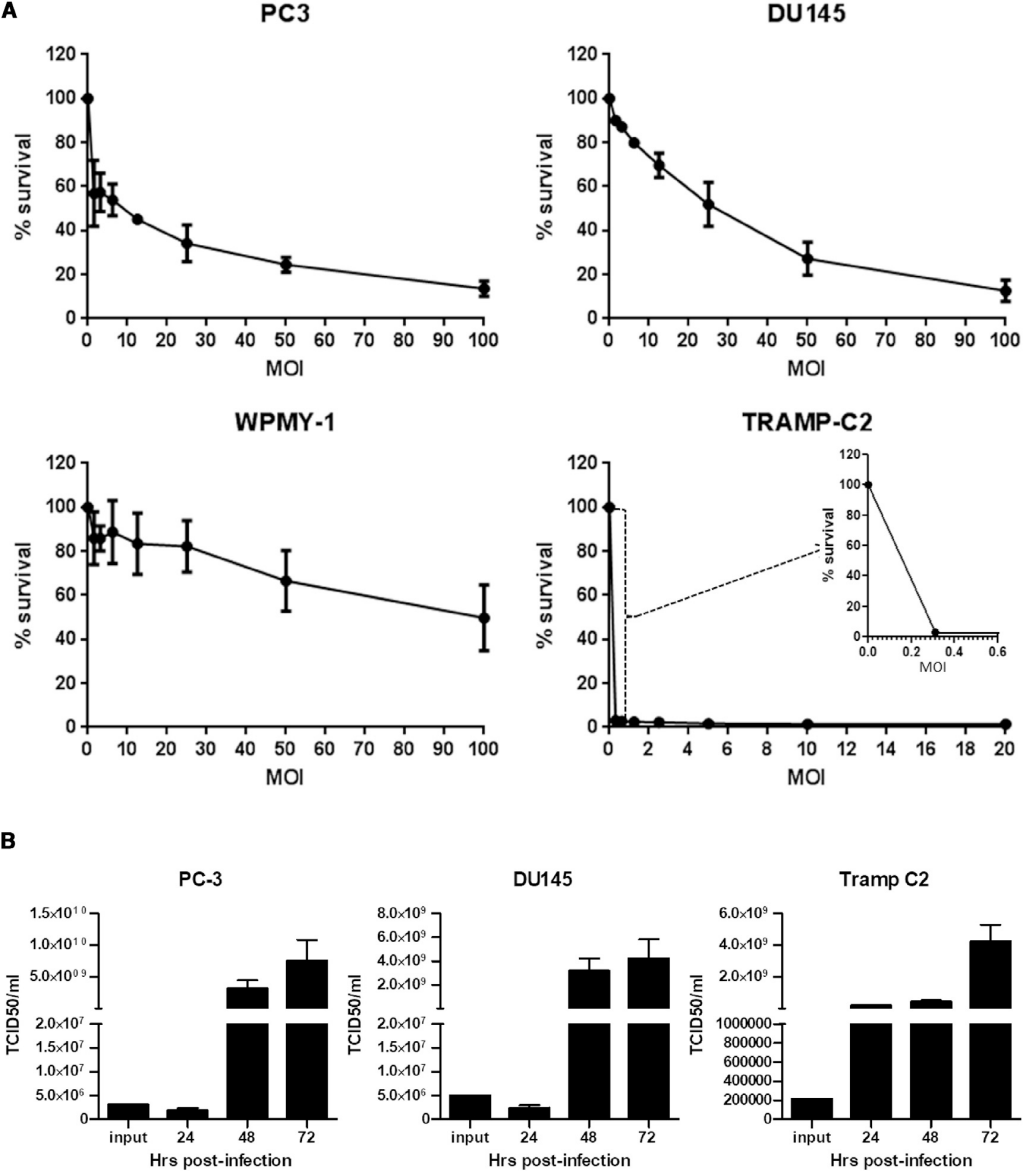
*In Vitro* Susceptibility to Reovirus Infection of a Panel of Prostate Cancer Cell Lines (A) Cell monolayers of human prostate cancer cell lines (PC3 and DU145), a human prostatic stromal myofibroblast cell line WPMY-1, and the transgenic adenocarcinoma mouse prostate cell line TRAMP-C2 were infected with doubling dilutions of a stock preparation of reovirus (3 × 10^9^ PFUs/mL). Following incubation at 37°C for 72 h, cell survival was determined by MTS assay. Data are presented as the average ± SD (n = 2). (B) Prostate cancer cell lines PC-3, DU145, and TRAMP-C2 grown in six-well tissue-culture plates were infected with reovirus at their MOIs at IC_50_, 3 for PC3, 40 for DU145, and 0.06 for TRAMP-C2, and incubated at 37°C for defined intervals. The culture media were harvested and centrifuged, and virus contained in the cell supernatants was titrated on L929 cell monolayers. TCID_50_ viral titers were calculated using the Spearman and Kärber algorithm as described in Hierholzer and Killington.^29^ Data are presented as the average ± SD (n = 2).

In order to use the TRAMP-C2-immunocompetent prostate cancer model for studies investigating the synergistic effect of combining reovirus oncolytic virotherapy with ICB, we first tested *in vitro* the susceptibility of TRAMP-C2 cells to reovirus infection and compared this with the known reovirus-susceptible prostate cell lines PC-3 and DU145. As depicted in Figure 1A, the TRAMP-C2 cell line was found to be highly susceptible to reovirus-induced oncolysis, even compared with the susceptible lines PC-3 and DU-145. The control cell line, WPMY-1 (a human prostatic myofibroblast cell line), remained largely refractory to infection by reovirus. These results are in keeping with those of Gujar et al.,^30^ who evaluated the status of activated Ras, known to be associated with the susceptibility of tumor cells to reovirus-mediated oncolysis,^31^^,^^32^ in a panel of prostate cancer cell lines and indeed showed that the TRAMP-C2-related cell line, TRAMP-C1, contained higher levels of activated Ras protein than PC-3 and DU145 cells, and thus a greater susceptibility to reovirus.

Following reovirus infection, all three prostate cancer cell lines, PC-3, DU145, and TRAMP-C2, produced a significant amount of progeny virus as compared with input viral titer in the culture supernatant by 72 h post-infection (Figure 1B), confirming the ability of reovirus to replicate in susceptible cell lines and release infectious progeny virus for subsequent infection cycles.

**Figure 2:**
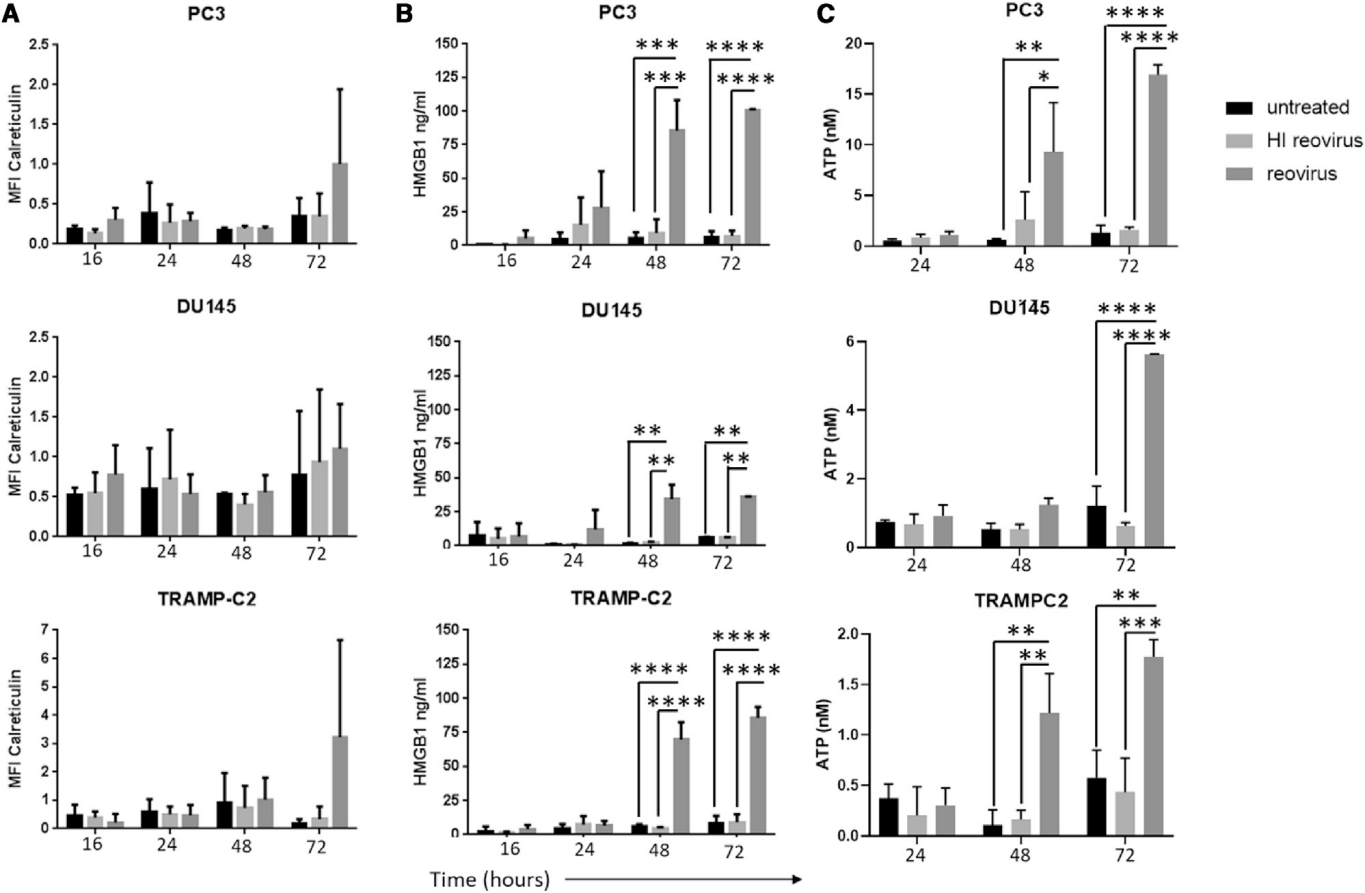
Induction of Immunogenic Cell Death Determinants in Response to Reovirus Infection in Prostate Cancer Cell Lines The human prostate cancer cell lines PC3 and DU145 and the mouse transgenic adenocarcinoma prostate cell line TRAMP-C2 were treated with reovirus at an MOI of 3 for PC3, 40 for DU145, and 0.06 for TRAMP-C2. (A) Cells were harvested at 16-, 24-, 48-, and 72-h time points, and flow cytometry was performed. The mean fluorescent intensity (MFI) of calreticulin-positive cells was gated on viable cells (ViViD-negative cells), thus detecting surface-exposed calreticulin rather than total calreticulin. Results are from two independent experiments (n = 2, mean ± SD). Supernatants were harvested at 16-, 24-, 48-, and 72-h time points. (B and C) Reovirus-triggered extracellular HMGB1 accumulation was determined by ELISA analysis of supernatants (B), and extracellular ATP accumulation was determined by a bioluminescence assay for quantitative determination of ATP (C) (significant differences between untreated or inactivated virus and reovirus-infected cultures as determined by two-way ANOVA; ∗∗p < 0.01,∗∗∗p < 0.001, ∗∗∗∗p < 0.0001). Results are from two independent experiments (n = 2, mean ± SD).

Given the well-recognized role of the immune response in the therapeutic efficacy of oncolytic viral therapy, the ICD profile of reovirus-infected PC3, DU145, and TRAMP-C2 cells was investigated. The cells were infected with reovirus MOIs at the relevant IC_50_s for each cell line, as determined above, and analyzed for expression/secretion of calreticulin, heat shock protein 70 (HSP70), HMGB1, and adenosine triphosphate (ATP) at various time points. The cell-surface expressed ICD determinants, calreticulin and HSP70, were analyzed by fluorescence-activated cell sorting (FACS), while the secreted determinants ATP and HMGB1 were assayed by a bioluminescence assay for quantitation of ATP and an HMGB1 ELISA, respectively. Although no specific induction of HSP70 was observed (Figure S1), reovirus infection of prostate cancer cell lines appeared to induce calreticulin translocation to the surface of the cells at 72 h after infection, although this did not reach statistical significance (Figure 2A). The induction of HMGB1 and ATP release were even more robust ICD features of reovirus infection, with the supernatants of reovirus-infected cultures containing significantly more HMGB1 and ATP than the untreated and inactivated reovirus-treated ones. Extracellular HMGB1 and ATP significantly increased from 48 h post-infection (Figures 2B and 2C).

**Figure 3:**
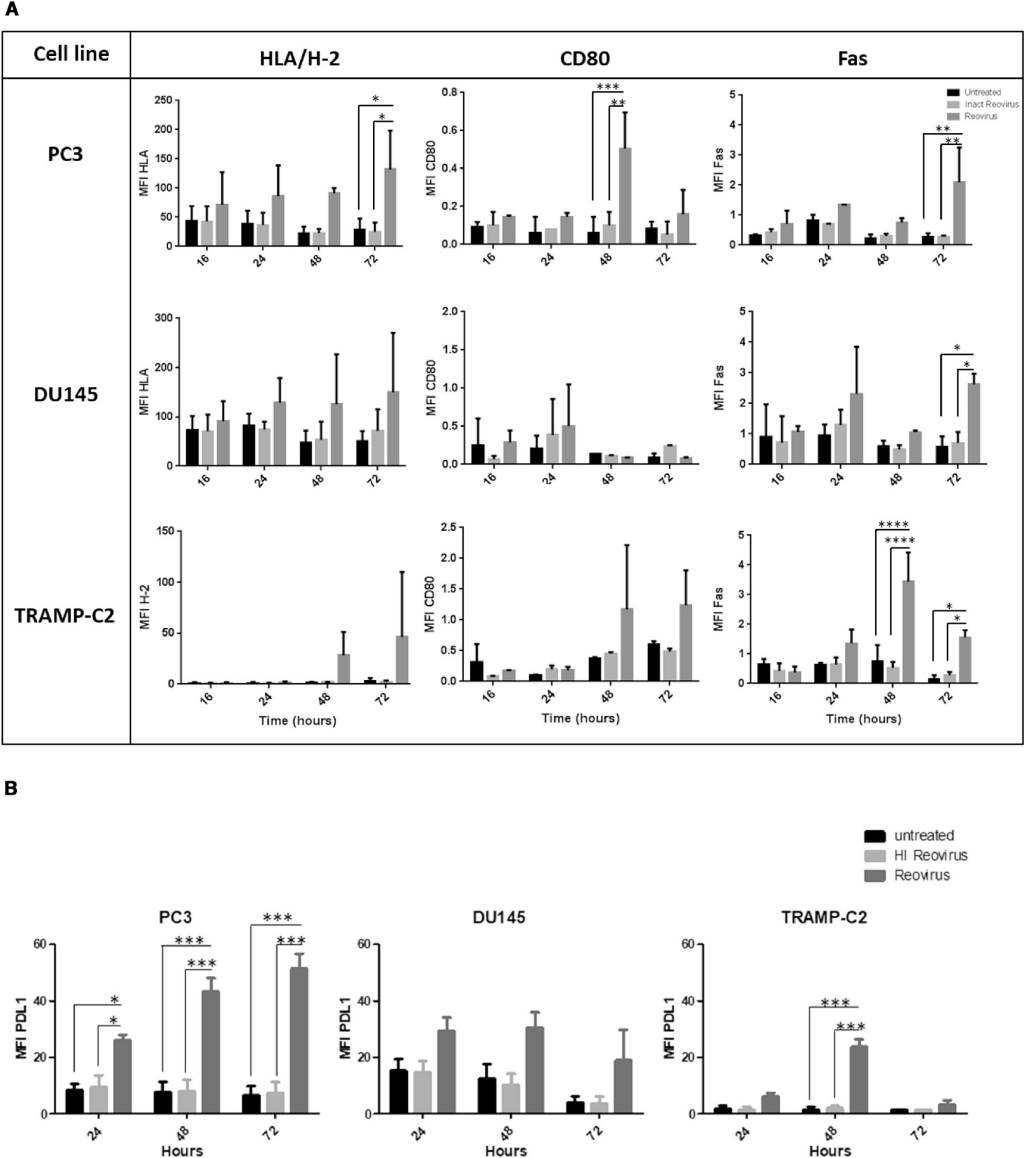
Reovirus Infection of Prostate Cancer Cell Lines Upregulates Cell Surface Molecules Associated with Susceptibility to Immune Attack The human prostate cancer cell lines PC3 and DU145 and the mouse transgenic adenocarcinoma prostate cell line TRAMP-C2 were treated with reovirus at an MOI of 3 for PC3, 40 for DU145, and 0.06 for TRAMP-C2. (A and B) Cells were harvested at 16-, 24-, 48-, and 72-h time points, and (A) HLA/H-2, CD80, Fas, and (B) PD-L1 expression were assessed by flow cytometry. Results are from two independent experiments (n = 2, mean ± SD). Significant differences between untreated or inactivated virus and reovirus-infected cultures were determined by two-way ANOVA; ∗∗p < 0.01, ∗∗∗p < 0.001, ∗∗∗∗p < 0.0001.

In addition, the potential of reovirus to influence the expression of cell surface molecules associated with susceptibility to immune attack was assessed. As shown in Figure 3A, reovirus infection caused an upregulation of HLA/H-2, CD80, and Fas, which was most evident in the PC3 and TRAMP-C2 cell lines. The induction of these proteins was most prevalent from 48 h post-reovirus infection. Thus, these *in vitro* data showing the ability of reovirus to infect prostate cancer cell lines and subsequently generate immunogenic tumor cell death supports the capability of reovirus to trigger a potent anti-tumor immune response.

As has been previously shown by our own work and others,^24^^,^^33^^,^^34^ OVs through their ability to induce local expression of type I interferons (IFNs) and type II IFN-γ result in the upregulation of inhibitory ligands (PD-L1 and PD-L2) on tumor cells, thereby making “cold” tumors susceptible to ICB. This was indeed shown to be the case for reovirus infection of both human and murine prostate cancer cell lines, which all demonstrated an increase in PD-L1 expression by 48 h post-virus infection, in keeping with the time taken to complete one life cycle of reovirus (between 18 and 24 h post-infection).^35^ In contrast, no increase in PD-L1 expression was observed for untreated or heat-inactivated reovirus-treated prostate cancer cell lines (Figure 3B).

### Targeted Blockade of Immunosuppressive Pathways following Reovirus Oncolytic Virotherapy Can Induce Synergistic Anti-tumor Responses

We initially tested a treatment regimen with reovirus in TRAMP-C2-bearing C57BL/6 immunocompetent mice, which would result in a suboptimal anti-tumor response. The intratumoral administration of reovirus (0.75 × 10^8^ plaque-forming units [PFUs]) on days 0, 2, and 5 delayed tumor growth but had no significant effect on survival compared with PBS-treated mice (Figure S2), and thus this regimen was taken forward for all subsequent combination *in vivo* experiments.

**Figure 4:**
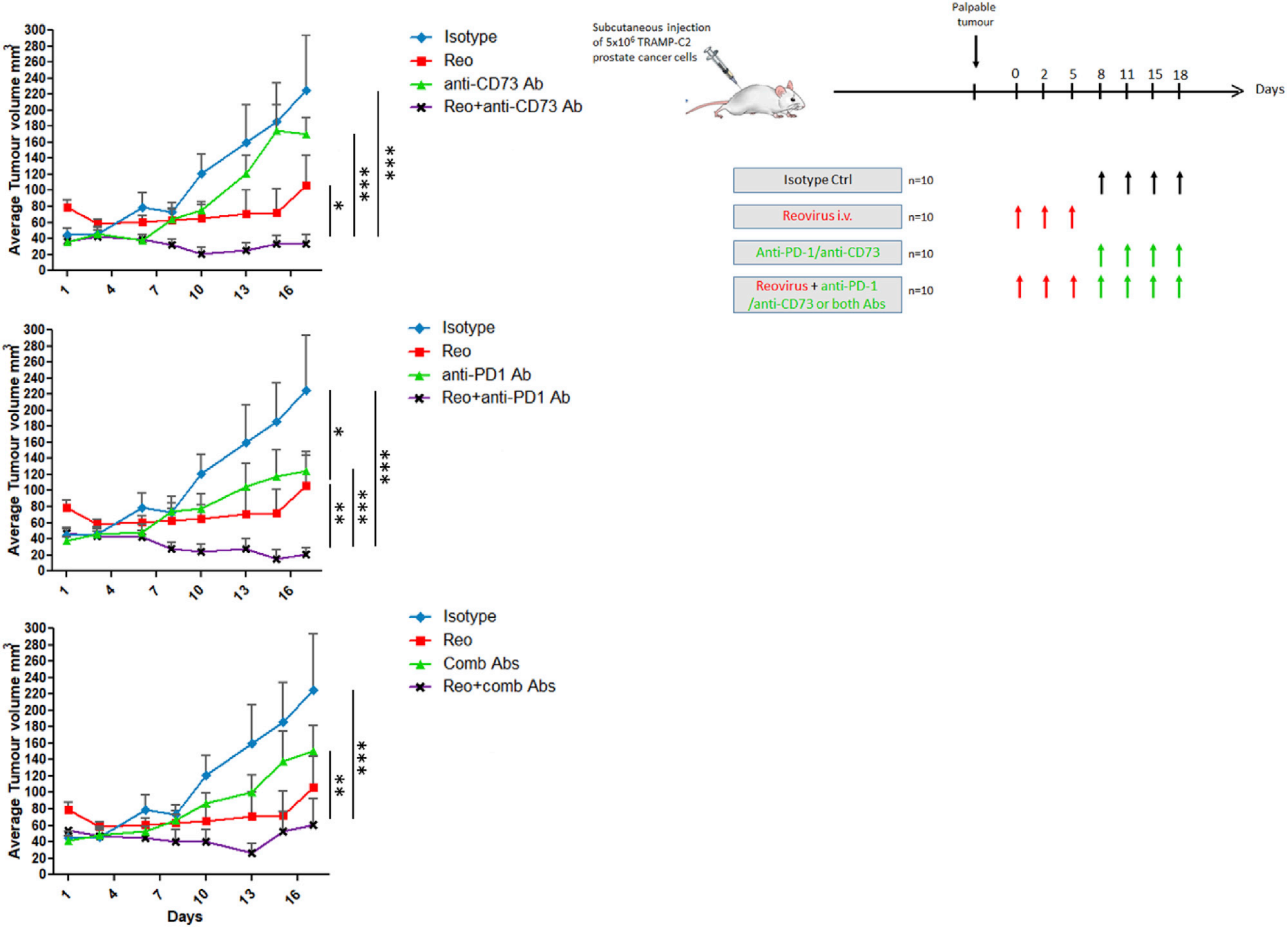
Reovirus Infection of Tumors Is Needed before a Therapeutic Effect of Anti-immune Inhibitory/Suppressive Antibodies Is Seen Anti-CD73 and anti-PD-1 alone or in combination was tested as a monotherapy, as well as in combination with reovirus infection. Mice bearing TRAMP-C2 tumors were first intratumorally administered with reovirus at 0.75 × 10^8^ PFUs on days 0, 2, and 5 before the addition of 100 μg anti-CD73, 100 μg anti-PD-1, or a combination of the antibodies twice weekly intraperitoneally. Control mice received 100 μg of isotype-control antibody. Tumor incidence and growth were monitored, and the statistical significance of the intergroup comparisons of tumor volumes was analyzed using two-way ANOVA; ∗p < 0.05; ∗∗p < 0.01; ∗∗∗p < 0.001.

As shown in Figure 4, twice weekly intraperitoneal treatment of TRAMP-C2-bearing C57BL/6 immunocompetent mice with either an anti-CD73 mAb (clone: TY/23; BioXCell) or an anti-PD-1 mAb (clone: RMP1-14; BioXCell) or the combination of both antibodies also gave no tumor control benefit. However, when anti-CD73 mAb or anti-PD-1 mAb or the combination of antibodies was administered 3 days following the last intrathecal (i.t.) reovirus treatment, combining treatments (reovirus plus antibody) significantly prolonged the tumor control of the mice beyond that observed with reovirus alone.

Complete tumor regressions were predominantly observed in animals treated with reovirus followed by mAb treatment (3/10 in the reo + anti-CD73 group, 4/9 in the reo + anti-PD-1 group, and 3/9 in the reo + combination antibody group). In contrast with previous findings in 3-methylcholanthrene (MCA)-induced fibrosarcomas,^28^ our results showed no enhancement of targeted blockade of CD73 to the therapeutic activity of anti-PD-1. Only 1/9 mice treated with reovirus alone had a complete regression, while the isotype control group and mAb-alone-treated groups did not result in any tumor regressions.

**Figure 5:**
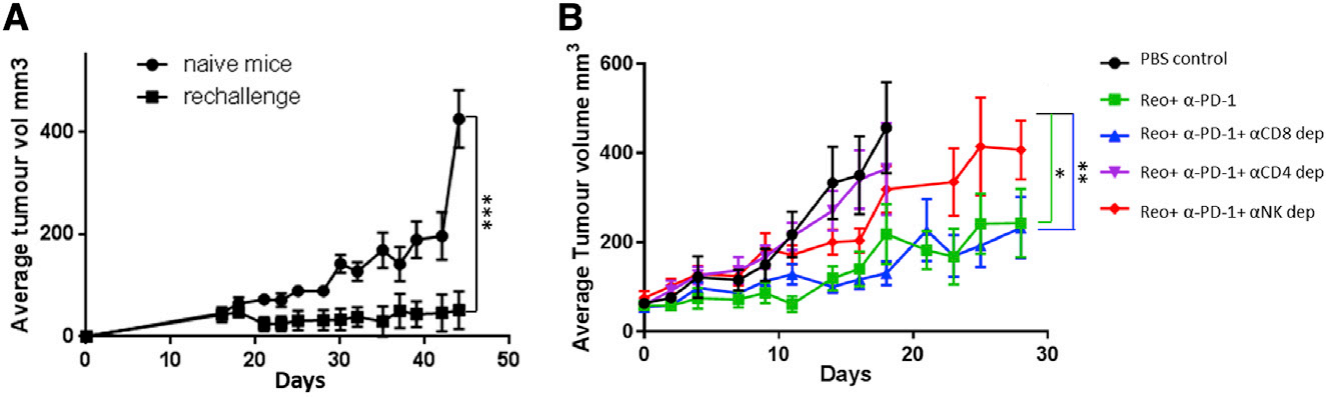
Reovirus-Initiated Anti-tumor Immunity Protects against Subsequent Tumor Challenge and Depends on CD4^+^ T Cells and NK Cells (A) C57BL/6 mice who had demonstrated complete remission of their tumors following reovirus plus antibody therapy were subsequently further challenged 63 days after initial viral therapy with 5 × 10^6^ TRAMP-C2 cells. A control group of naive C57BL/6 mice also received 5 × 10^6^ TRAMP-C2 cells. Both groups of mice were monitored for tumor growth. (B) C57BL/6 mice were treated as described in Figure 4 with or without depleting antibodies for CD4^+^, CD8^+^, or NK cells and monitored for tumor growth. The statistical significance of the intergroup comparisons of tumor volumes was determined using a two-way ANOVA. ∗p < 0.05; ∗∗p < 0.01; ∗∗∗p < 0.001.

In order to investigate the clinical significance of the immune responses observed in the previous experiment, we evaluated the capacity of reovirus immunotherapy-induced anti-tumor T cell responses to protect against subsequent tumor challenge. For this purpose, mice who had demonstrated a complete regression with any of the reovirus plus antibody combinations were re-challenged, 63 days after initial viral therapy, with 5 × 10^6^ TRAMP-C2 cells. Compared with the control group of naive mice who upon challenge with TRAMP-C2 cells quickly developed tumors, the re-challenged group of previously treated reovirus/ICB mice did not develop tumor outgrowth (Figure 5A).

To determine which immune cell type(s) was responsible for the therapeutic efficacy of oncolytic reovirus treatment, we examined its effect on tumors in mice treated with neutralizing antibodies against CD8, CD4, and NK. For this experiment we concentrated on the reovirus plus anti-PD-1, which had given the best therapeutic outcome. Adequate cell depletion of each cell subset was confirmed by flow cytometry of splenocytes (data not shown). As shown in Figure 5B, depletion of CD8^+^ T cells did not result in any appreciable change in anti-tumor effect, whereas depletion of either CD4^+^ T cells or NK cells resulted in abrogation of the therapeutic effect in virus-injected tumors.

These results demonstrate that reovirus immunotherapy-initiated anti-tumor innate and adaptive immune cell response can restrict the growth of freshly implanted tumor cells. These data also show that, once appropriately activated, anti-tumor responses act in a reovirus-independent manner as the protection against tumor challenge was achieved without administering secondary reovirus injection.

### Genes Involved in Cytokine-Cytokine Receptor Interaction, Immune Cell Recruitment, and Immune Cell Regulation Are Significantly Increased in Reovirus-Treated TRAMP-C2 Tumors

**Figure 6:**
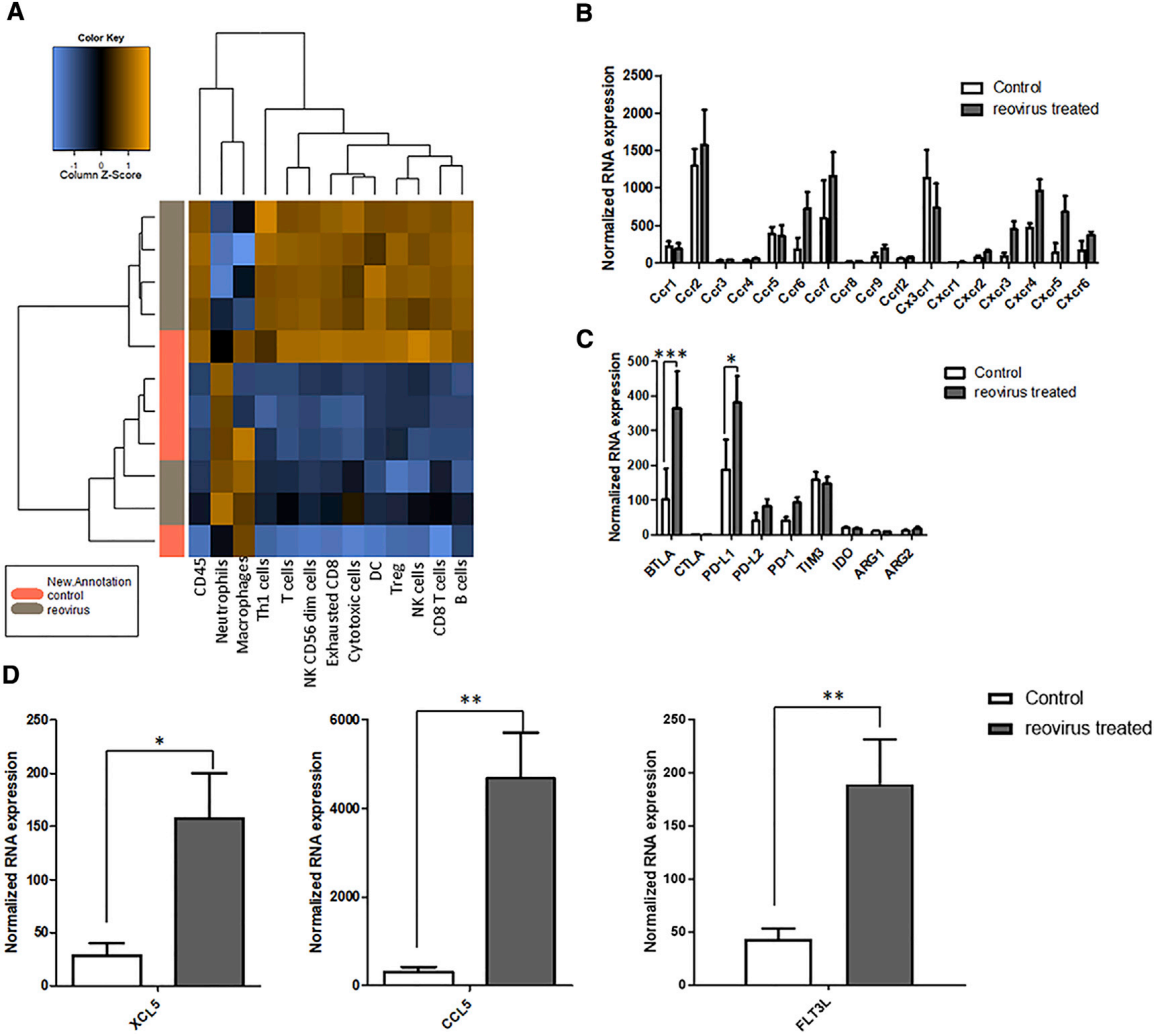
Reovirus Infection of Tumors Induces Significant Expression of Immune Subset Genes, Immune-Regulatory Genes, and Stimulatory Dendritic Cell Chemoattractants NanoString’s Pan Cancer Immune Profiling RNA Panel was used to investigate the differential gene expression of total RNA from untreated or reovirus-treated TRAMP-C2 tumors. (A) A heatmap showing the expression of genes encoding for different innate and adaptive immune cell subsets were significantly increased in the reovirus-treated tumors compared with the control PBS-treated tumors. (B–D) Genes encoding chemokine receptors (B), the immune-regulatory genes PD-L1 and BTLA (C), and chemoattractants for stimulatory DCs (XCL5, CCL5, and FLT3LG) (D) were significantly increased in the reovirus-treated tumors compared with untreated tumors (significant differences between untreated or reovirus-infected cultures as determined by unpaired t test; ∗p < 0.05, ∗∗p < 0.01,∗∗∗p < 0.001).

In order to assess the impact of reovirus therapy and understand the synergistic effect with immune-modulating antibodies, we used NanoString’s PanCancer Immune Profiling RNA Panel to investigate the differential gene expression within the control untreated and reovirus-treated TRAMP-C2 tumors. This analysis focused on genes expressed by immune cell types, their effector molecules, and any negative regulators that may impede the clinical effectiveness of reovirus-induced immune responses. As shown in Figure 6A, deconvolution of the NanoString data to identify cell types clearly demonstrated the ability of reovirus infection to cause an increase in innate (NK cells and DCs) and adaptive immune cell types (T cells and B cells) within the virus-treated TRAMP-C2 tumors as compared with untreated tumors. This recruitment of specific immune cell types was in keeping with the increased chemokine receptor expression (CCR6, CCR7, and CXCR3 involved in recruitment/activation of T cells, NK cells, and DCs, and CXCR5 involved in B cell migration) observed within the reovirus-treated tumors. Of note, within the reovirus-treated tumors, there was a significant upregulation of genes involved with NK and DC functions, early innate immune cells crucial to the initiation of an immune response (Figure S3). In particular, the observed recruitment of DCs in reovirus-treated tumors correlated with the significant upregulation of genes encoding XCL5, CCL5, and FLT3LG, known chemoattractants for stimulatory DCs (sDCs), a cell type integrally important for immune responses to cancer and responsiveness to anti-PD-1 immunotherapy.

Although the use of OVs such as reovirus can overcome pre-existing mechanisms of resistance to ICB in prostate cancers by transforming these cold tumors into “hot,” immune cell-infiltrated tumors, such biological therapy may be further enhanced with the use of relevant ICB that can overcome any constitutive or compensatory inhibitory resistance mechanisms. Thus, genes encoding negative regulators were analyzed in the untreated and reovirus-treated TRAMP-C2 tumors. Of the panel of negative regulators studied, only *BTLA* and *PD-L1* were significantly upregulated in the reovirus-treated TRAMP-C2 tumors compared with untreated tumors (Figure 6C).

**Figure 7:**
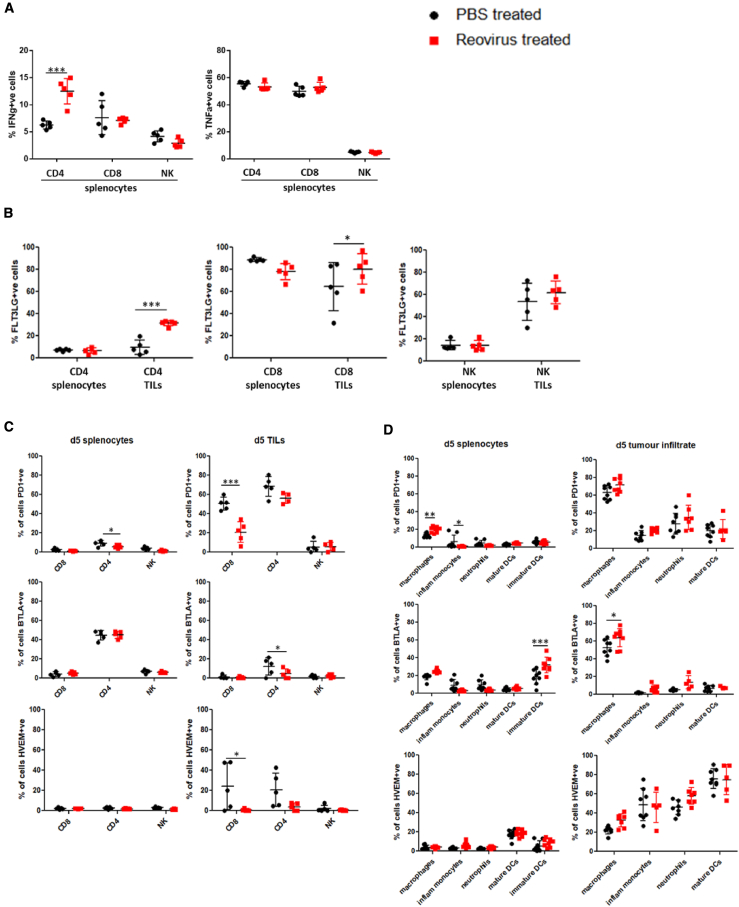
Functional and Cell Surface Expression Characterization of Immune Cell Populations from Reovirus-Treated versus Untreated Mice TRAMP-C2 tumors harvested 5 days after intratumoral reovirus or PBS treatment were subjected to both mechanical and enzymatic dissociation using the gentleMACS Dissociator, while spleens harvested from the same mice were mechanically disrupted to obtain splenocyte cell suspensions. (A–D) The resulting tumor single-cell suspensions and/or splenocytes were then immuno-phenotyped to characterize (A) the different lymphoid immune populations for their functional ability to produce cytokines upon *in vitro* stimulation and for their expression of (B) FLT3LG, (C and D) PD-1, BTLA, and HVEM by (C) lymphocytes (CD8^+^, CD4^+^, and NK cells) and (D) innate inflammatory cell subsets (macrophages [F4/80^+^], inflammatory monocytes [Ly6Chi, CD11b^+^], neutrophils [Ly6G^+^, CD11b^+^], mature DCs [CD11c^+^, MHC II^+^], and immature DCs [CD11c^+^, MHC class II^−^]). Significant differences between immune cell populations derived from untreated or reovirus-infected tumors was determined using a two-way ANOVA; ∗p < 0.05, ∗∗p < 0.01, ∗∗∗p < 0.001.

To further confirm and understand the role of particular immune genes induced by reovirus treatment within the TRAMP-C2 tumor microenvironment, we performed another experiment using mice implanted with TRAMP-C2 tumors and treating them with or without reovirus (as in previous experiments). Mice from the two groups were sacrificed 5 days following reovirus treatment, and their splenocytes and TILs were obtained for further characterization. In keeping with the cell depletion experiment showing a prominent role for CD4^+^ T cells in the therapeutic outcome, this analysis revealed that there was a significantly larger percentage of IFN-γ-expressing cells only within the CD4^+^ splenocyte population from reovirus-treated mice compared with PBS control-treated mice (Figure 7A). Furthermore, although the expression of the cytokine FLT3LG appeared to be constitutively high on CD8^+^ T cells and NK cells within tumors compared with splenocytes, regardless of whether they had been treated with reovirus or not, the expression of FLT3LG was most significantly increased on the CD4^+^ TIL population from reovirus-treated tumors (Figure 7B).

Although blockade of the PD-L1 signal with an anti-PD-1 antibody clearly sensitized TRAMP-C2 tumors to cytotoxic T lymphocyte (CTL) killing in the current study, we were interested to understand the role of the significant BTLA upregulation observed in reovirus-treated tumors by NanoString analysis. In contrast with the PD-1/PD-L1 inhibitory interaction, BTLA interaction with its ligand HVEM, rather than acting as an inhibitory interaction, may under certain conditions transmit positive co-signals into effector T cells that promote their survival. Thus, surface expression of BTLA and its ligand HVEM were analyzed on lymphocytes (CD4^+^, CD8^+^ T cells, and NK cells), as well as on innate cell populations derived from the spleens and tumors of mice treated with or without intratumoral injection of reovirus. As shown in Figure 7C, the expression of BTLA was consistently low on lymphocytes derived from the spleens and tumors of both treated and untreated mice. This was in contrast with PD-1 expression, which was high on both tumor-infiltrating CD4^+^ and CD8^+^ T cell subsets. We did, however, observe high expression of BTLA predominantly on macrophages (F4/80^+^ cells) within the innate inflammatory cell populations studied from the tumors of mice, with a higher expression of BTLA^+^ macrophages noted in the reovirus-treated tumors (Figure 7D). Interestingly, although HVEM was constitutively expressed at low levels on the T cells and NKs from the spleens of treated and untreated mice, HVEM was expressed at higher levels on both CD8 and CD4 T cells in the tumors of uninfected mice. However, after infection with reovirus, both the CD8^+^ and CD4^+^ T cells had downregulated HVEM (Figure 7C), in keeping with previous reports showing the downregulation of HVEM on virus-specific T cells at the acute stage of vaccinia virus infection.^36^ HVEM was also highly expressed by the majority of innate inflammatory cell types infiltrating the tumors. To understand whether the increased BTLA expression observed on innate inflammatory cell tumor infiltrate and its corresponding changes in ligand expression on T cells in response to virus infection was not unique to this model, BALB/c mice bearing CT26 tumors were similarly treated with intratumoral administration of reovirus on three consecutive time points and sacrificed 5 days post-treatment as before. As shown in Figure S4, the TILs displayed similar levels of PD-1 and BTLA expression, except the NK cells, which had high levels of PD-1 expression in the BALB/c:CT26 model, were not observed in the C57BL/6:TRAMP-C2 model. HVEM expression on the lymphocytes was similar in both models in the untreated tumors, but the downregulation of HVEM within the CD4^+^ T cell subset was not observed in the BALB/c:CT26 model. Within the innate inflammatory cell tumor infiltrate, no expression of BTLA was observed on the macrophages. However, there was high expression of BTLA on the CD11c^+^ DCs. These cell-type differences in BTLA expression in various murine backgrounds have been reported before.^37^

## Discussion

This study using oncolytic reovirus in combination with ICB adds to the growing evidence that the combination of OV and ICB is an effective therapy for re-sensitizing immune checkpoint-resistant tumors into immunotherapy-responsive tumors.^38^, ^39^, ^40^ A number of clinical studies with PD-1 inhibitors in combination with a number of DNA and RNA OVs are ongoing.^41^^,^^42^ Early insights have been gained in the successful combination of ipilimumab and T-VEC,^43^ and a further study of the same virus in combination with pembrolizumab is ongoing (keynote-034, ClinicalTrials.gov: NCT02263508; https://clinicaltrials.gov/ct2/show/NCT02263508). Such an approach holds real promise for tumors such as prostate cancers that have, to date, been shown to be largely insensitive to immune checkpoint inhibitors, with only 5% of prostate cancer patients responding in a recent checkpoint inhibitor clinical trial.^44^

The ability of OVs to sensitize immunologically cold tumors to ICB is due to their potent ability to inflame tumor microenvironments with both innate and adaptive immune cells. This was clearly demonstrated in this study, with reovirus infection of TRAMP-C2 tumors resulting in the recruitment of both innate and adaptive immune effectors. The importance of this inflammatory ability of OVs has been given further weight by recent data showing that the early recruitment of particular innate immune cells is critical to initiate an anti-tumor immune response and subsequent response to immune checkpoint inhibitors.^45^, ^46^, ^47^, ^48^ In particular, NK-sDC interactions that exist in special anatomical locations within tumors are uniquely associated with responsiveness to anti-PD-1 immunotherapy.^48^ Furthermore, it was found that NK cells are the integral cell type that produce FLT3LG to control the levels of these sDCs in tumors. We were able to demonstrate significantly increased levels of both NK and DC function genes, as well as chemoattractants known to attract sDCs in reovirus-treated tumor tissues. One of these chemoattractants, FLT3LG, appeared to be due to both NK and T cells, with CD4^+^ T cells showing the greatest increase in response to reovirus treatment. The importance of FLT3LG for anti-tumor responses in murine cancer models has already been shown in numerous publications,^48^^,^^49^ including a study by Tourkova et al.^50^ that demonstrated the important role of NK production of FLT3LG for protection/survival of DC precursors from prostate cancer-induced inhibition *in vivo*.

Unlike many previous oncolytic virotherapy studies, this study using reovirus in a TRAMP-C2 model demonstrated a clear dependence on both NK and CD4^+^ T cells for the therapeutic efficacy of this treatment. Although the dependence on NK cells could be because of their early role in recruiting sDCs to initiate anti-tumor immunity, the demonstration that the CD4^+^ T cells were the most potent producers of IFNg in response to reovirus infection supports their direct role in control of tumor growth. This induction of a strong OV-induced CD4 T cell response has been demonstrated before in a breast cancer mouse model treated with a targeted replicating recombinant vesicular stomatitis virus (rrVSV). In this model the CD4 T cells responsible for the immune memory response were highly potent and could support a strategy of dealing with the problem of cancer metastases by immunoprevention.^51^ In addition, previous work from our own group demonstrated the dependence on a Coxsackievirus A21-induced CD4 response to control the outgrowth of MB49 bladder tumors in mice.^52^

One notable finding from this study was the upregulation of BTLA expression in reovirus-infected tumors. Although BTLA has been implicated in contributing to many disease states, including cancer,^53^ because of its ability to inhibit the adaptive immune response, recent studies have now shown that BTLA may also, in the context of a live virus infection, transmit positive co-signals into effector T cells that promote their survival.^36^ This was elegantly shown by Flynn et al.^36^ using an experimental vaccinia virus infection in mice. They showed that HVEM- and BTLA-deficient mice have a severe defect in mounting a protective CD8 T cell response against vaccinia virus. Furthermore, HVEM expression on CD8^+^ T cells and BTLA expression by a different cell type was necessary for continued survival of virus-specific effector CD8 T cells and optimal generation of memory. This function of BTLA as a *trans*-activating ligand delivering pro-survival signals through HVEM expressed on T cells has also been proposed for HVEM during infection with the intracellular bacteria *Listeria monocytogenes*.^54^ Because we found similar kinetics of HVEM expression on T cells derived from reovirus-infected tumors as observed during acute vaccinia virus infection,^36^ and high expression of BTLA on macrophages or CD11c^+^ DCs within two reovirus-treated tumor models studied, we believe that the HVEM-BTLA trans co-signaling system could be contributing to the functional outcomes of responding T cells and development of the protective immune response observed in the reovirus-treated mice. The emerging evidence for the critical role of the HVEM-BTLA *trans* co-signaling system in antiviral immunity could be used to determine or even augment OV-induced anti-tumor immune responses in the future. However, further work using *BTLA*^−/−^ or *HVEM*^−/−^ mice is needed to confirm the role that HVEM:BTLA interactions have in response to OV therapy.

In summary, we believe the results from this study further add to the evidence that OV therapy provides the best way to attract and activate early innate effectors, such as NK cells and antigen-presenting cells (APCs), within the TME, which are essential for initiating anti-tumor immunity, thus explaining why initial OV therapy of prostate tumors is crucial to obtain a subsequent therapeutic outcome from ICB.

## Materials and Methods

### Reovirus

The Reovirus Type 3 Dearing (T3D) (Reolysin) was obtained from Oncolytics Biotech, Canada. Virus stock titer and virus stability were measured by standard plaque assay of serially diluted samples on L929 cells. Heat inactivation of the reovirus was performed by heating 200 μL aliquots of viral stock at 3 × 10^9^ PFUs/mL for 20 min at 60°C.

### Cell Cultures and Treatments

The prostate cancer-derived cell lines PC-3, DU145 (human), and TRAMP-C2 (Murine) and the human prostatic stromal myofibroblast cell line were obtained from American Type Culture Collection (ATCC). All cell lines were authenticated by DDC Medical with short tandem repeat profiling (STR) in 2014 and stocks frozen. Cell lines were checked monthly using MycoAlert PLUS Mycoplasma Detection Kit (Lonza, Switzerland). The PC-3, DU 145, and WPMY-1 cell lines were routinely grown in F-12K, Eagle’s minimal essential medium (EMEM), and DMEM, respectively, all supplemented with 10% fetal bovine serum (FBS), penicillin/streptomycin, and glutamine. The TRAMP-C2 cell line was maintained in DMEM supplemented with 5% heat-inactivated FBS, 5% Nu-Serum IV (Corning), 10 mg/L bovine insulin (Sigma-Aldrich, UK), and 0.01 mM dihydrotestosterone (DHT) (Fisher, UK).

### *In Vitro* Effects of Reovirus on Cell Viability

The prostate cancer cell lines were plated in 96-well flat-bottom plates in media and incubated at 37°C overnight before treatment with media alone (control wells), heat-inactivated reovirus, or live reovirus. The reovirus infection was conducted at a starting MOI of 100 for PC-3, DU 145, and WPMY1 and 20 for TRAMP-C2 with 2-fold viral dilutions. After 72 h, survival in each treatment was assessed by the (3-(4,5-dimethylthiazol-2-yl)-5-(3-carboxymethoxyphenyl)-2-(4-sulfophenyl)-2H-tetrazolium) (MTS) colorimetric cell viability assay (CellTiter 96 Aqueous One Solution Cell Proliferation Assay; Promega), relative to untreated cells.

### Production of Viral Progeny

Cell culture supernatants of samples from prostate cancer cell lines infected with reovirus (MOIs at half maximal inhibitory concentration [IC_50_], 3 for PC3, 40 for DU145, and 0.06 for TRAMP-C2) were collected and stored at −80°C until analysis. Viral progeny titers were measured by the 50% tissue culture dose (TCID_50_) assay on L929 cells and calculated by the Spearman and Kärber algorithm as described by Hierholzer and Killington.^29^

### ICD Determinant Analysis

FACS analysis was used to determine expression of ICD determinants on the surfaces of reovirus-treated cells. Cell lines untreated, treated with reovirus (MOIs at IC_50_, 3 for PC3, 40 for DU145, and 0.06 for TRAMP-C2), or exposed to heat-inactivated reovirus for 24, 48, and 72 h were incubated with anti-HSP70 (clone: EPR16892), anti-calreticulin (rabbit polyclonal), and a rabbit isotype control (Abcam, UK). Additional stains included major histocompatibility complex (MHC) class I (clone: W6132; BioLegend, UK), CD80 (clone: 2D10; eBioscience, UK), FAS (rabbit polyclonal; Abcam, UK), and CD274 (PD-L1) (M1H2; BioLegend, UK). Relevant secondary Alexa 488-labeled antibodies were subsequently applied (Molecular Probes, UK). For the detection of murine markers on the TRAMP-C2 line, the additional following antibodies were used: anti-mouse H-2 (clone: M1/42; BioLegend, UK), anti-mouse CD80 (clone: 16-10A1; BioLegend, UK), and anti-mouse CD274 (PD-L1) (clone: M1H7; eBioscience, UK).

Released ATP and HMGB1 levels in cell supernatants of cell lines untreated, treated with reovirus, or exposed to heat-inactivated reovirus for 24, 48, and 72 h were detected using a standard ATP determination kit according to the manufacturer’s (Molecular Probes) instructions or measured by an HMGB1 ELISA (IBL International, Hamburg, Germany).

### Tumor Models and Treatment Regimens

*In vivo* procedures were approved by the UK Home Office (License no. PBE74785E) and by Animal Welfare Ethical Review Body (AWERB) of the University of Surrey. All experiments conformed to all relevant regulatory standards. Six- to seven-week-old, male immunocompetent C57BL/6 mice were obtained from Envigo (Huntingdon, UK). The immunocompetent C57BL/6 mice were implanted with 5 × 10^6^ TRAMP-C2 cells in one flank subcutaneously on day 0. Once palpable tumors were established, the TRAMP-C2 tumor-bearing mice were divided into four groups of mice (n = 10 mice per group) and treated with 100 μg isotype antibody (Rat IgG2a, clone 2A3; BioXCell) intraperitoneally twice weekly for 2 weeks alone, reovirus alone (0.75 × 10^8^ PFUs given intratumorally on days 0, 2, and 5), 100 μg anti-CD73 (clone TY/23; BioXCell), or 100 μg anti-PD-1 (clone RMP1-14; BioXCell) alone administered from day 8 intraperitoneally twice weekly for 2 weeks or reovirus followed by anti-CD73 or anti-PD-1 combination. In a further experiment, TRAMP-C2 tumor-bearing mice were again divided into four groups and treated as above with isotype antibody alone, reovirus alone, or the combination of both anti-CD73 and anti-PD-1 or reovirus followed by the combination of antibodies. Tumor size was measured and recorded twice weekly.

For tumor re-challenge studies, C57BL/6 TRAMP-C2 tumor-bearing mice successfully treated with reovirus-antibody combination therapy were re-challenged with 5 × 10^6^ TRAMP-C2 cells in the contralateral hind flank, and tumor burden was assessed by caliper measurement. Treatment-naive C57BL/6 mice were challenged in the same manner to serve as a control.

For the cell depletion study, antibodies against CD4 (100 μg clone GK1.5; BioXCell, USA), CD8 (100 μg clone 2.43; BioXCell, USA), or NK (100 μg clone PK136; BioXCell, USA) were injected intraperitoneally every 3 days depending on the dosing schedule.

### RNA Extraction from Tumors

RNA was isolated from harvested TRAMP-C2 tumors using a gentleMACS Dissociator system (Miltenyi Biotec, Germany) with M tubes (Miltenyi Biotec, Germany) and the RNA_02 program recommended by the manufacturer. After homogenization, the isolation of RNA was performed using a RNeasy Plus Mini Kit (QIAGEN, UK). The resulting concentration and quality of the RNA were assessed using an Agilent 2100 Bioanalyzer System.

### NanoString

Digital multiplexed NanoString nCounter analysis system (NanoString Technologies, Seattle, WA, USA)-based gene expression profiling was performed on 100 ng total RNA from untreated or reovirus-treated TRAMP-C2 tumors according to the manufacturer’s instructions. NanoString RNA analysis of 700 immune-related genes was performed using the nCounter GX Murine PanCancer Immune profiling Kit (XT) on the nCounter Analysis System. Analysis and normalization of the raw NanoString data were performed using nSolver Analysis Software v.1.1 (NanoString Technologies).

### Mouse Tumor Dissociation and Immune Profiling

Harvested TRAMP-C2 tumors were subjected to both mechanical and enzymatic dissociation using the gentleMACS Dissociator in combination with the Tumor Dissociation Kit, mouse (Miltenyi Biotec, UK). Spleens were also harvested from the same mice, and splenocyte cell suspensions were obtained by mechanical disruption. The resulting tumor single-cell suspensions and splenocytes were then immuno-phenotyped to characterize the different immune populations. The following fluorochrome-labeled antibodies for surface staining were used: anti-CD45 (clone: REA737; Miltenyi Biotec), anti-CD4 (clone: GK1.5; BD Biosciences), anti-CD8 (clone: 53-6.7; BD Biosciences), anti-NK1.1 (clone: PK136; Miltenyi Biotec), anti-BTLA (clone: 8F4; BioLegend), anti-PD-1 (clone: REA802; Miltenyi Biotec), anti-HVEM (clone: LH1; Invitrogen), anti-FLT3LG (goat IgG; R&D Systems, UK), anti-F4/80 (clone: BM8; Invitrogen), anti-CD11b (clone: M1/70; eBioscience), anti-Ly6C (clone: REA796; Miltenyi Biotec), anti-Ly6G (clone: REA526; Miltenyi Biotec), anti-MHC class II (clone: M5/114.15.2; Miltenyi Biotec), and anti-CD11c (clone: HL3; BD Biosciences).

Intracellular cytokine staining was performed by stimulating splenocytes or TILs for 6 h with Ionomycin (Sigma UK) and PMA (Sigma UK). After 2 h, 10 μg/mL brefeldin A (Sigma UK) was added. Following the stimulation, cells were fixed and permeabilized using the Fix and Perm kit (Thermo Fisher, UK) before staining for cytokines by the addition of anti-IFN-γ (clone: XMG1.2; eBioscience, UK) and anti-TNF-α (clone: REA636; Miltenyi Biotec) fluorescently labeled antibodies. Analysis was performed using a MACSQuant flow cytometer (Miltenyi Biotec) and MACSQuantify software (Miltenyi Biotec).

## Author Contributions

N.E.A. acquired funding and devised experiments. N.E.A., G.R.S., M.A., and M.D. conducted experiments. M.C. provided virus in kind, academic input, and data analysis. H.P., A.M., K.H., and R.V. provided academic advice and guidance and proofread the manuscript. N.E.A. wrote and edited the manuscript.

## Conflicts of Interest

M.C. is President and Chief Executive Officer at Oncolytics Biotech. All authors declare no competing interests.

## Supplemental Information

Document S1. Figures S1–S4

Document S2. Article plus Supplemental Information
