# Supplementary material for: Oncolytic Reovirus-Mediated Recruitment of Early Innate Immune Responses Reverses Immunotherapy Resistance in Prostate Tumors
Source: Mol Ther Oncolytics. 2020 Oct 4;20:434–46. doi: 10.1016/j.omto.2020.09.010 (PMC7900644; doi:10.1016/j.omto.2020.09.010)
Supplement: Document S1. Figures S1–S4 [file mmc1.pdf]

OMTO, Volume *20*

Supplemental Information

Oncolytic Reovirus-Mediated Recruitment of Early Innate Immune Responses Reverses Immunotherapy Resistance in Prostate Tumors

Nicola E. Annels, Guy R. Simpson, Mick Denyer, Mehreen Arif, Matt Coffey, Alan Melcher, Kevin Harrington, Richard Vile, and Hardev Pandha

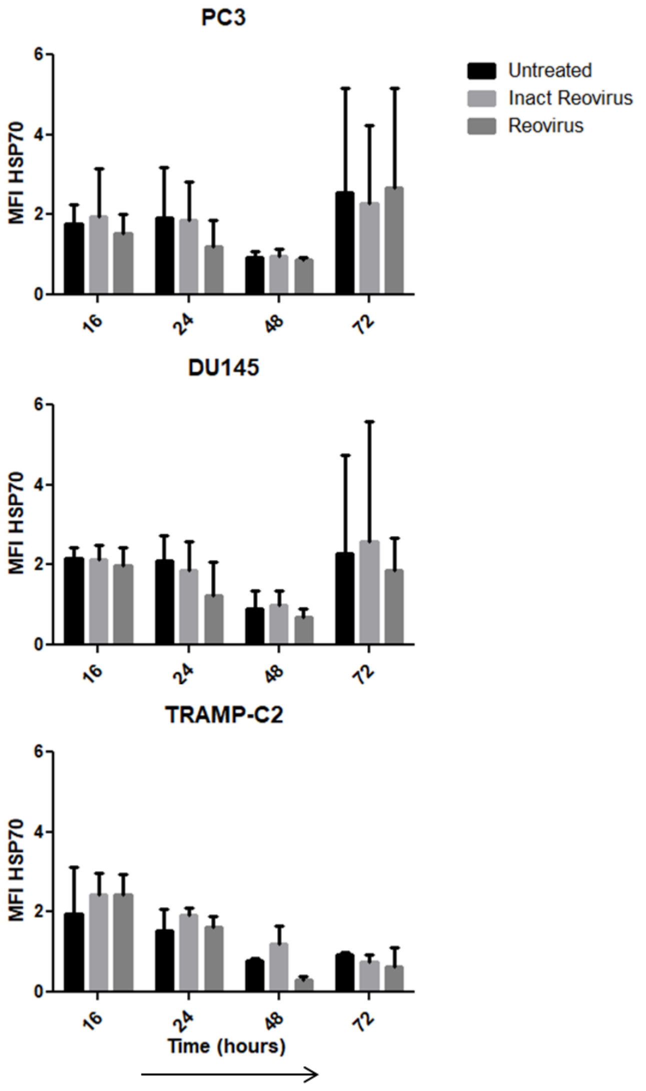

Figure S1: No induction of immunogenic cell death determinant, HSP70, in response to reovirus infection in prostate cancer cell lines

The human prostate cancer cell lines PC3 and DU145 and the mouse transgenic adenocarcinoma prostate cell line TRAMP-C2 were treated with reovirus at an MOI of 3 for PC3, 40 for DU145 and 0.06 for TRAMP-C2. (A) Cells were harvested at 16, 24, 48 and 72hour time-points and flow cytometry for HSP70 was performed. The mean fluorescent intensity (MFI) of HSP70 on the different prostate cancer cell lines in response to no treatment, inactivated reovirus, or reovirus is shown. Results are from two independent experiments (n=2, mean ± SD).

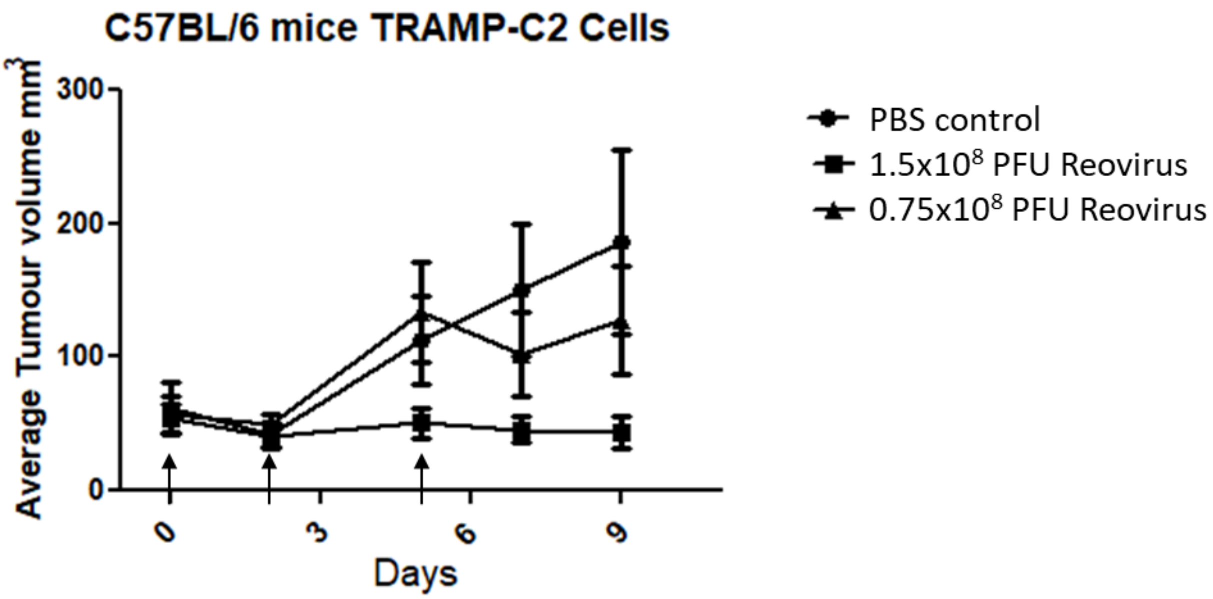

Figure S2: Susceptibility of mouse transgenic adenocarcinoma of mouse prostate-C2 (TRAMP-C2) cells to reovirus in vivo.

Immunocompetent C57BL/6 mice were subcutaneously implanted with $5x10^6$ TRAMP-C2 cells and evaluated for tumour growth. When tumours reached ~$50mm^3$, mice were intratumorally administered with $1.5x10^8$pfu or $0.75x10^8$pfu of reovirus or PBS at the time points indicated by arrows, and then monitored for tumour growth.

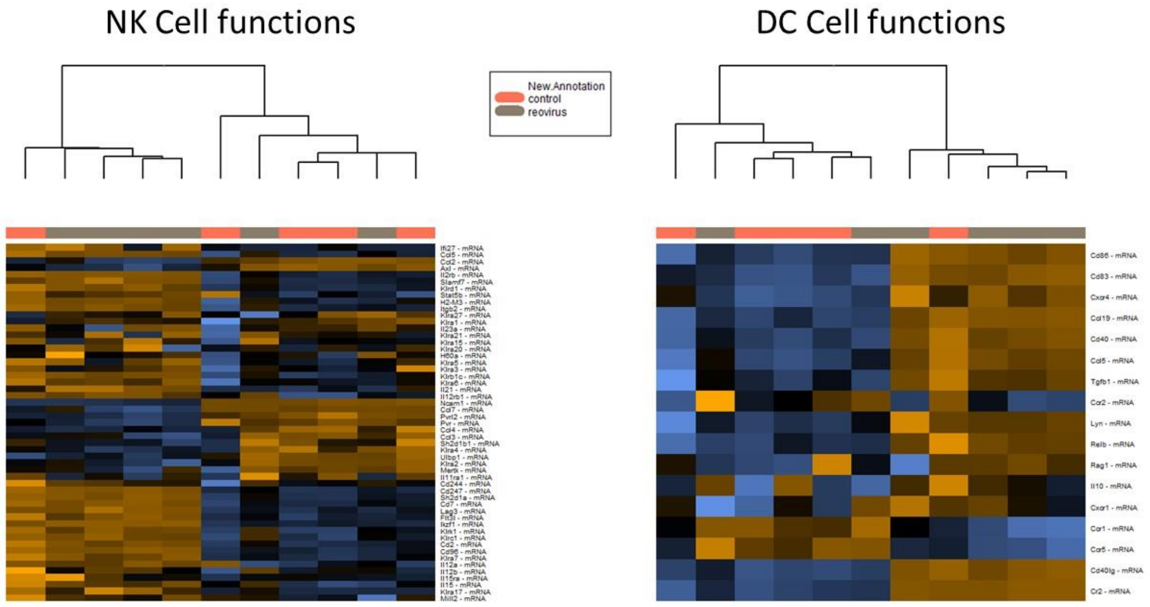

Figure S3: Increase in early innate effector function genes in reovirus-treated tumours

Nanostring's Pan Cancer Immune Profiling RNA Panel was used to investigate the differential gene expression of total RNA from untreated or reovirus-treated TRAMP-C2 tumours. The heatmaps show that the expression of genes related to NK or DC cell functions were significantly increased in the reovirus-treated tumours compared with the control PBS-treated tumours.

A

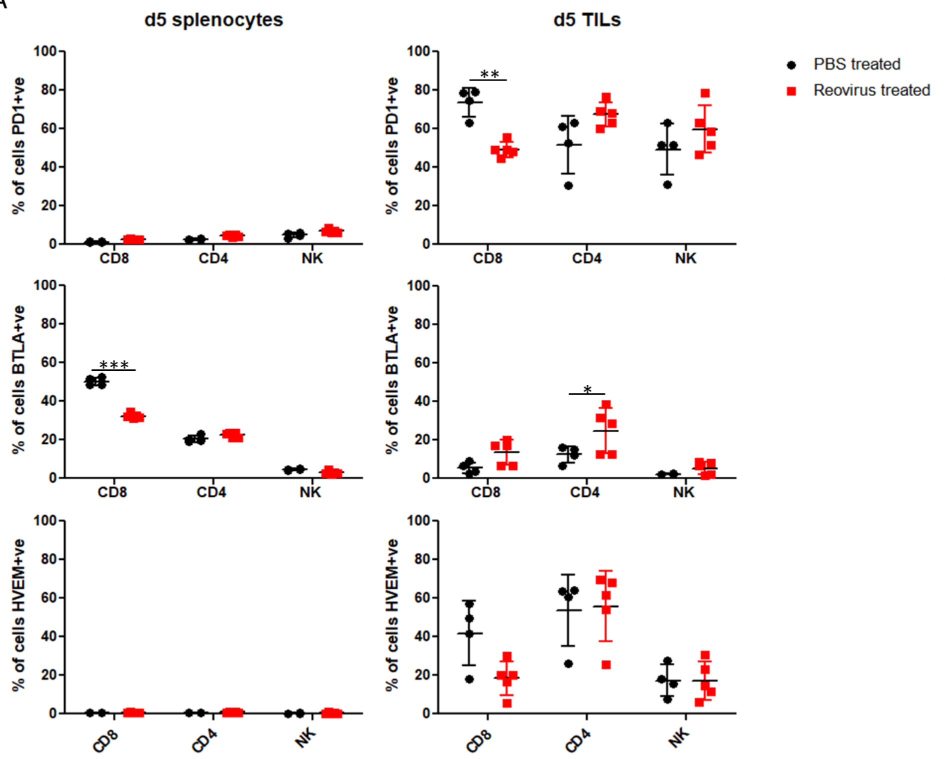
d5 splenocytes
d5 TILs
PBS treated
Reovirus treated
% of cells PD1+ve
% of cells BTLA+ve
% of cells HVEM+ve
CD8
CD4
NK
**
***
*

B

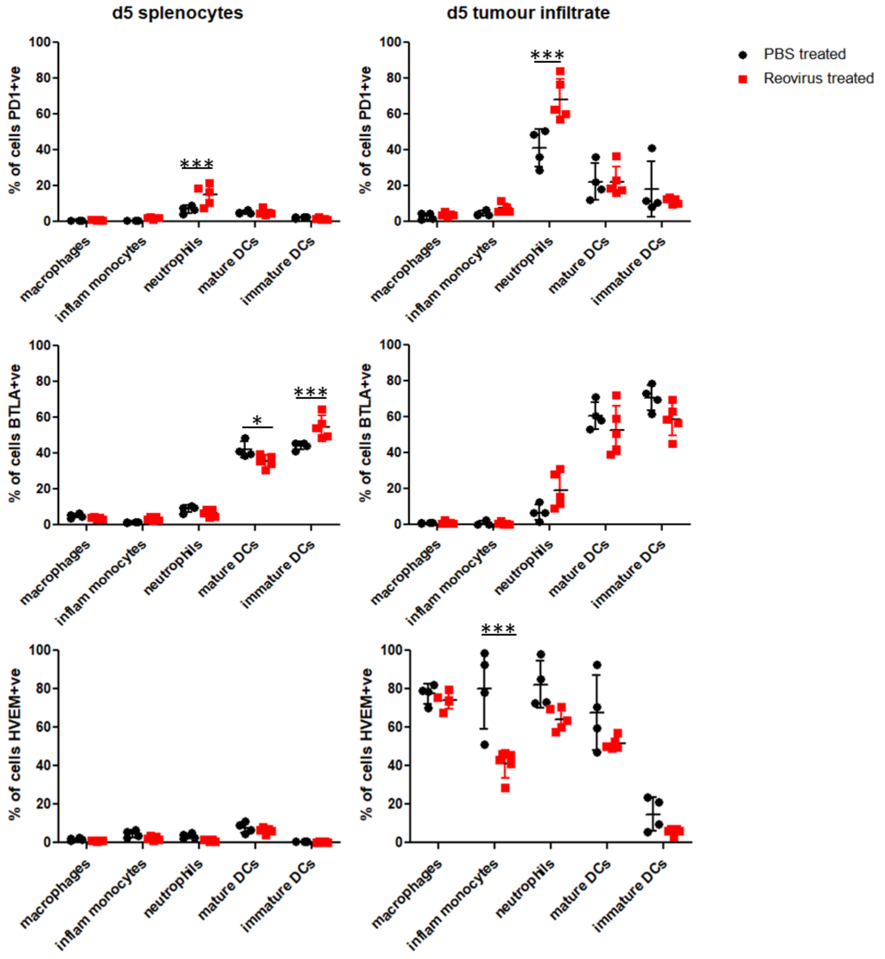
d5 splenocytes
d5 tumour infiltrate
PBS treated
Reovirus treated
% of cells PD1+ve
% of cells BTLA+ve
% of cells HVEM+ve
macrophages
inflam monocytes
neutrophils
mature DCs
immature DCs
***
*

Figure S4: Expression of PD1, BTLA and HVEM on innate and adaptive cell subsets in the BalbC:CT26 tumour model

CT26 tumours harvested 5 days after intratumoural reovirus or PBS treatment were subjected to both mechanical and enzymatic dissociation using the gentleMACS™ Dissociator whilst spleens harvested from the same mice were mechanically disrupted to obtain splenocyte cell suspensions. The resulting tumour single-cell suspensions and/or splenocytes were then immuno-phenotyped to characterise PD1, BTLA and HVEM expression on (A) lymphocytes and (B) innate inflammatory cell subsets (Macrophages (F4/80+), inflammatory monocytes (Ly6Chi, CD11b+), neutrophils (Ly6G+, CD11b+), mature DCs (CD11c+, MHC II+), and immature DCs (CD11c+, MHC II−)). Significant differences between immune cell populations derived from untreated or reovirus-infected tumours was determined using a two-way ANOVA; * $p<0.05$, ** $p<0.01$,*** $p<0.001$).
